# Supplementary material for: The Modular Architecture of Metallothioneins Facilitates Domain Rearrangements and Contributes to Their Evolvability in Metal-Accumulating Mollusks
Source: Int J Mol Sci. 2022 Dec 13;23(24):15824. doi: 10.3390/ijms232415824 (PMC9781358; doi:10.3390/ijms232415824)
Supplement: Supplementary file 1 [file ijms-23-15824-s001.zip › suplementary-material.pdf]

SUPLEMENTARY MATERIAL

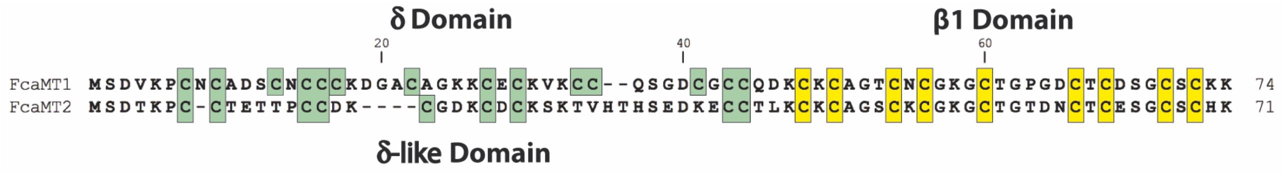

Supplementary Figure S1. Amino acid alignment of *Falcidens caudatus* MTs displaying a bidomain structure, δβ1 for FcaMT1 and δ-likeβ1 for FcaMT2. Cysteines are highlighted in yellow for the β1 domain, and in green for the δ (above) and δ-like (below).
